# Mammalian gastrointestinal tract parameters modulating the integrity, surface properties, and absorption of food‐relevant nanomaterials

**DOI:** 10.1002/wnan.1333

**Published:** 2015-01-30

**Authors:** Susann Bellmann, David Carlander, Alessio Fasano, Dragan Momcilovic, Joseph A. Scimeca, W. James Waldman, Lourdes Gombau, Lyubov Tsytsikova, Richard Canady, Dora I. A. Pereira, David E. Lefebvre

**Affiliations:** ^1^ TNOUtrechtThe Netherlands; ^2^ Nanotechnology Industries AssociationBrusselsBelgium; ^3^ Massachusetts General Hospital for ChildrenHarvard Medical SchoolBostonMAUSA; ^4^ Department of Health and Human ServicesUS Food and Drug AdministrationSilver SpringMDUSA; ^5^ Cargill, IncorporatedMinneapolisMNUSA; ^6^ Ohio State UniversityColumbusOHUSA; ^7^ Leitat Technological CenterBarcelonaSpain; ^8^ Center for Risk Science Innovation and ApplicationILSI Research FoundationWashingtonDCUSA; ^9^ MRC Human Nutrition ResearchElsie Widdowson LaboratoryCambridgeUK; ^10^ Regulatory Toxicology Research DivisionFood Directorate, Health CanadaOttawaCanada

## Abstract

Many natural chemicals in food are in the nanometer size range, and the selective uptake of nutrients with nanoscale dimensions by the gastrointestinal (GI) tract is a normal physiological process. Novel engineered nanomaterials (NMs) can bring various benefits to food, e.g., enhancing nutrition. Assessing potential risks requires an understanding of the stability of these entities in the GI lumen, and an understanding of whether or not they can be absorbed and thus become systemically available. Data are emerging on the mammalian in vivo absorption of engineered NMs composed of chemicals with a range of properties, including metal, mineral, biochemical macromolecules, and lipid‐based entities. In vitro and in silico fluid incubation data has also provided some evidence of changes in particle stability, aggregation, and surface properties following interaction with luminal factors present in the GI tract. The variables include physical forces, osmotic concentration, pH, digestive enzymes, other food, and endogenous biochemicals, and commensal microbes. Further research is required to fill remaining data gaps on the effects of these parameters on NM integrity, physicochemical properties, and GI absorption. Knowledge of the most influential luminal parameters will be essential when developing models of the GI tract to quantify the percent absorption of food‐relevant engineered NMs for risk assessment. *WIREs Nanomed Nanobiotechnol* 2015, 7:609–622. doi: 10.1002/wnan.1333

For further resources related to this article, please visit the WIREs website.

## INTRODUCTION

Many chemicals naturally found in the environment, in food, and in the human body are in the nanometer (nm) size range.^1^ For example, many globular proteins have a diameter of several nanometres, starch granules have substructures of approximately 30 nm, the DNA double helix has a diameter of 2 nm, and fatty acids are several nanometers in length.^2^, ^3^ Traditional food processing practices such as emulsification also generate nanosized structures such as micelles, foams, and colloids in the food matrix and many such materials have a long history of safe consumption; for example, micelles created during the homogenization of milk. Apart from food origins, there are endogenous nanoparticles (NPs) physiologically produced from ions in the mammalian gastrointestinal (GI) tract. For example, the precipitation of calcium and phosphate creates nanosized calcium phosphate particles that may be absorbed as such.^4^ Thus, the presence and uptake of nutrients with nanoscale dimensions is a normal physiological process.

Scientific innovation is expanding the diversity of approaches used to produce food to meet the needs of the global population. Nanotechnology, an enabling technology with applications in many divergent sectors, is being explored within the food arena to bring beneficial properties to food products and enhance nutrition. Consequently, it is necessary to develop scientific tools for the detection and risk assessment of novel engineered nanomaterials (NMs) / NPs in food.^5^ The NanoRelease Food Additive project, coordinated by the International Life Sciences Institute (ISLI) Research Foundation, is aiming to identify and advance NM measurement methods to support international risk assessment capacity and safe product development for engineered NMs in food. The actionable conclusions synthesized by the task groups of this project are summarized in a State of the Science report.^6^

A task group of the NanoRelease Food Additive project first reviewed the types of engineered NMs that may potentially be used in food.^7^ The presence of these substances could arise from direct incorporation during food manufacturing or from migration from food contact materials. NMs could also be incidentally present in food from environmental sources. Three broad categories of potential food‐relevant examples were defined as (1) soft/lipid‐based, such as solid lipid NPs, (2) solid non‐lipid non‐metal, such as silicon dioxide (SiO_2_), carbon black or cellulose NMs, and (3) solid metalloid / metal‐based, such as titanium dioxide (TiO_2_) or silver NPs.^7^ Another group of the project reviewed analytical methods that can be used to detect NMs in complex food and food contact material matrices, and their release from these matrices.^8^, ^9^, ^10^, ^11^

Following the consumption of a chemical entity, the percentage or total mass absorbed to systemic circulation can be used, along with other toxicology data, as a key parameter in the determining safe maximum levels in food.^12^ Therefore the NanoRelease Food Additive project also reviewed existing models of the GI tract that could be adapted to allow the assessment of digestion and bioavailability.^13^ The analysis concluded that *in silico* computational, *in vitro* fluid, and *in vitro* cell culture assays should be used, after which the necessity of *ex vivo* organ and *in vivo* animal models should be considered.

This article from the NanoRelease project summarizes GI conditions influencing the absorption of NM entities *in vivo*, including but not limited to engineered metal, mineral, carbohydrate, nucleic acid, protein, and lipid nanostructures. Along with a general review and examples relevant to those broad categories we have included some specific examples of TiO_2_, SiO_2,_ and cellulose NMs. These three substances were selected as examples because a survey of the multistakeholder NanoRelease steering committee determined that they may have potential uses or presence in food, reflect a range of material characteristics including release potential and solubility, are of interest to various stakeholders, and are being considered for further efforts to develop measurement methods. TiO_2_, also known as titanium dioxide or titania, is an oxide of the transition metal element titanium. Its bulk anatase and rutile crystal forms are used as a coloring agent in food.^7^ SiO_2_, also termed silicon dioxide or silica, is an oxide of the metalloid element silicon. It is used as an anticaking agent in food. Cellulose is a biochemical polymer consisting of glucose monomers, and is a major structural component of plants, bacteria, and algae. Bulk cellulose and microcrystalline cellulose are used as anticaking agents in food.^14^ These chemicals can all be engineered down to nanoscale structures, and can remain intact when suspended in aqueous media or added to dry food matrices.^15^, ^16^ Examples of other pertinent chemicals are also discussed below. We reviewed uptake and evaluated evidence of whether or not the various physicochemical conditions encountered in the GI tract modify NP size and surface properties. This knowledge will be important when developing models to quantify the absorption of novel engineered NMs for risk assessment.

## GI TRACT ANATOMY MEDIATING THE ABSORPTION OF NMs

### Macroscopic and Microscopic Anatomy

**Figure 1:**
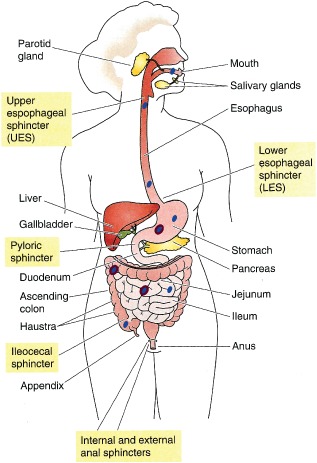
The transit of consumed particulates through the lumen of the organs of the human digestive system. The buccal cavity, esophagus, stomach, small intestine, and large intestine are separated from each other by sphincters, labeled in beige squares. Consumed particulates (shown in blue) passing through these organs may or may not remain in their native physicochemical state, and can develop a dynamic corona coating (represented in violet). (Reprinted with permission from Ref 18. Copyright 2009 Elsevier)

The mammalian GI tract spans from the mouth to the anus, with an average length of approximately 5 m in humans.^17^ The macroscopic compartments, separated from each other by sphincters, are the buccal cavity, esophagus, stomach, small intestine, and large intestine (Figure 1). The primary functions are the maintenance of water homeostasis, the digestion, and absorption of macro‐ and micronutrients and electrolytes, trafficking of the fraction of macromolecular antigens that survive digestion, and the exclusion of pathogens.

The continuous multilayer stratified squamous epithelium in contact with the lumen of the buccal cavity and esophagus is adapted to handle the high volume of quickly passing food. The surface area is 0.02 meters squared (m^2^) in the buccal cavity and likewise 0.02 m^2^ in the esophagus.^17^ There is little published information on the absorption rate of particulates through the epithelium of these two compartments. This is likely because the surface area is low, the residence time for most food matrices is short, and the intestine is more specialized for selective uptake of macromolecules in this size range. In the stomach the surface area is 0.05 m^2^. Despite the relatively low surface area and permeability of the gastric epithelium to macromolecules, a minimal passage of NPs is allowed.^19^, ^20^

**Figure 2:**
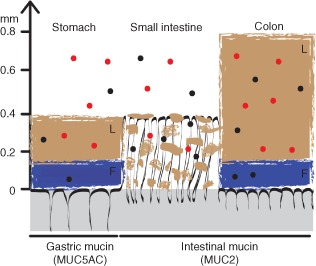
Mucus organization and nanoparticle interactions in the gastrointestinal tract. The gastrointestinal tissue is represented in grey with black folds representing the structure interfacing the lumen. The predominant mucin isotype expressed in each region is shown in parenthesis. L denotes the loosely bound outer mucus layer. Non‐interacting lamellar strands of loosely bound mucus in the small intestine are also shown in brown. F denotes the firmly attached inner mucus layer, shown in blue. Mucoadhesive nanoparticles are represented by the red circles; non‐mucoadhesive nanoparticles are represented by the black circles. (Reprinted with permission from Ref 22. Copyright 2011 National Academy of Sciences)

Before particles contact epithelial cells, they must first cross the mucus barrier. Mucus is comprised of mucin glycoproteins that form viscoelastic gels resulting in an adherent unstirred layer coating the GI wall.^21^ In the stomach and large intestine the mucus layer is tightly bound (Figure 2). Adhesive substances are trapped, but non‐adherent materials can diffuse through this layer. In the small intestine the mucus layer is thinner and there is less interaction between lamellar strings, allowing greater access of the luminal contents to the epithelium.^23^, ^24^ This permits the uptake of nutrients, while trapping, immobilizing, and excluding larger potentially hazardous particulates, e.g., bacteria.^25^

The small intestine consists of three consecutive parts: the duodenum, jejunum, and ileum. It is the longest segment of the GI tract, and the outermost microscopic layer is structured with villi and microvilli that project into the lumen, resulting in a very high surface area of 30 m^2^ in humans.^17^ In normal homeostasis the epithelial cell monolayer that covers the villi forms a tight but selective barrier. Microbes and most macromolecular antigens are held at bay, whereas nutrients are absorbed efficiently. The epithelial monolayer contains several specialized cell types. Enterocyte cells are responsible for nutrient absorption, while other cell types perform functions such as the secretion of mucus.^26^ The large intestine (colon) has haustral folds, but is shorter and lacks the villi projections seen in the small intestine, and therefore has a lower surface area of 2 m^2^. Columnar enterocytes in the epithelium are the predominant cell type.

Gut‐associated lymphoid tissue (GALT), distributed in localized regions of the wall of the small and large intestine, is composed of isolated follicles and of aggregated lymphoid follicles termed Peyer's patches. These tissues have a specialized epithelium containing antigen sampling microfold (M)‐cells, in addition to enterocytes, and have a much thinner mucus layer allowing direct interfacing with lumen contents.^27^ Overall, M‐cells represent approximately 1% of the cells lining the intestine.^28^ Functionally they transport particulate matter from the gut lumen across the epithelial barrier to allow sampling by antigen‐presenting cells of the immune system, which traffic through the extracellular lymph fluid on the basolateral side.^29^, ^30^

### Transepithelial Absorption Mechanisms

**Figure 3:**
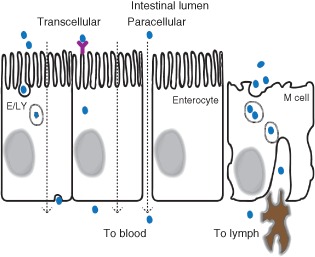
Pathways of nanoparticle absorption through the gastrointestinal tract epithelium. From left to right: vesicular endocytosis through epithelial cells where E/LY denotes endosome or lysosome; receptor‐mediated transport through epithelial cells; paracellular transport between epithelial cells; and vesicular phagocytosis through microfold (M) epithelial cells covering lymphoid aggregates, with dendritic cells below in brown. Nanoparticles are shown in blue.

Key to understanding NM absorption in the GI tract is an understanding of the various microscopic routes by which particulates can be taken up. Two transepithelial routes can allow the passage of substances from the lumen to the basolateral side: transcellular transport (i.e., through an epithelial cell) or paracellular transport (i.e., between adjacent epithelial cells; Figure 3).

Transcellular transport can be bi‐directional. When a substance is absorbed into a cell's cytoplasm through the apical membrane it can have several fates. It can (A) be secreted back into the lumen, (B) remain in the cell and accumulate, (C) degrade in the cell, or (D) be transported across the basolateral cell membrane into systemic circulation. There is limited simple diffusion of particulates into cells due to factors such as steric hindrance and immiscible solubility.^28^ Carrier‐mediated transport takes up specific small molecule ligands, and so is not specific to larger NMs. Vesicular transcytosis is the most efficient mechanism by which intact macromolecules can be transported into and/or across epithelial cells.^31^, ^32^ The process requires energy expenditure by the cells and, for many types of NMs, is dependent upon dynamic interactions of particles with the actin cytoskeleton, microfilaments, and microtubules.^33^ There are several types of vesicular transcytosis. Receptor‐mediated endocytosis begins when ligands in the contents of the intestinal lumen bind to receptor proteins (e.g., clathrin or caveolin) on the exterior of the apical cell membrane, triggering endocytosis. Because of the low endocytic activity of enterocytes, the amount of NPs translocated via this route is considered to be low.^28^ Nonetheless, endocytosis has been reported for NMs such as ferritin metalloprotein and tartrate‐modified ferrihydrite.^34^, ^35^, ^36^ Pinocytosis and macropinocytosis are non‐selective mechanisms of endocytosis. The resulting intracellular vesicles fuse with lysosomes for enzymatic degradation of the contents. Phagocytosis is the endocytosis of larger solid particles such as viruses, bacteria, or particulate matter. Lymphoid follicle and Peyer's patch M‐cells use both phagocytosis and receptor‐mediated endocytosis to sample lumen contents. It is generally believed that the majority of particle translocation through the intestinal wall occurs via this route.^28^, ^30^, ^37^

Paracellular transport is the passive movement of solutes across the epithelium between the tight junctions that bind cells together into a monolayer. The dimension of this paracellular space is on the order of nanometers and it varies along the intestinal tract.^38^, ^39^ In the esophagus and stomach, the epithelial barrier effectively restricts the paracellular movement of solutes, with a paracellular pore diameter of approximately 0.6 nm.^40^ In the small intestine the diameter is approximately 1 nm.^41^ However, the proximal duodenum region can permit tightly regulated permeation of molecules as large as albumin, which has a diameter of 7 nm.^40^ The large intestine has intermediate properties, allowing regulated passage of molecules of approximately 3 nm diameter.^42^ These properties are altered in several pathological circumstances (e.g., inflammation, erosion, radiation insult, etc.) in which transepithelial permeability through the paracellular pathway may be enhanced. Under normal conditions the paracellular route is generally not accessible to larger compounds. However, the normal process of persorption, whereby dead enterocytes are extruded from the epithelial layer, can create breaches in the barrier through which particulates can pass. This mechanism is unlikely to be a highly efficient pathway of NM uptake.^4^, ^43^ Nonetheless, it has been shown that 4 and 10 nm colloidal gold particles are absorbed through dead enterocytes being extruded from the intestinal villus.^44^

In summary, the major pathways implicated in the uptake of intact NPs across the GI wall are transcellular transport through viable M‐cells lining intestinal Peyer's patches and through the abundant intestinal enterocytes. However, degradation products released from the surface of NPs can also be absorbed by conventional mechanisms. This is important because, for some NM classes, ions released and absorbed into the body can reconstitute into NMs in tissues.^45^

Following the uptake of intact and degraded substances through epithelial cells to the underlying lymph fluid, they can either remain there, circulate in the lymph fluid through lymphatic ducts that eventually drain into the systemic cardiovascular circulatory system, or be absorbed directly into capillaries of the cardiovascular circulatory system.^46^ Buccal cavity and esophageal capillaries lead into systemic veins, whereas capillaries from the stomach and intestines pass through the liver before entering systemic circulation.^47^ A portion of absorbed substances can thereby be secreted back into the intestinal lumen via enterohepatic circulation through the gallbladder in bile. The remainder is distributed to systemic tissues and either metabolized in those tissues, excreted by other routes such as urine, or otherwise remains persistent.^48^ GI tissues, systemic blood and lymph, and peripheral tissues are examined to quantify the absorption of a consumed NM.

### Examples of NM Percent Absorption

There is a limited number of published *in vivo* mammalian studies that quantified GI absorption following consumption of the chemical entities we selected as examples of potentially food‐relevant NMs. While the number of studies might not yet be sufficient to allow an exact numerical conclusion, they suggest that only a low percentage of administered TiO_2_ and SiO_2_ NPs are absorbed into the circulating blood and peripheral tissues in mammals. The vast majority is excreted in the feces.

Percent absorption measurements were exemplified in rats orally exposed to 500 nm rutile TiO_2_ particles by gavage for 10 days.^49^ Following necropsy, microscopy visualized particles in the intestinal Peyer's patch. Spectroscopic analysis of titanium quantified that 0.06% of the administered dose was taken up by the stomach, 0.11% was in the small intestine, 4% was in the large intestine, (and of that 2.86% was in the Peyer's patches and lymphoid tissue), and 0.02% accumulated in the blood.^49^ Similarly in mice 6 h after a single gavage exposure to agglomerates of 12 nm anatase TiO_2_ NPs, some titanium was imaged inside sections of Peyer's patches and in the regular epithelium of the small intestine.^50^ The titanium was observed both in the cytoplasm and below the cells. Therefore it had been absorbed. Passage of either particles and/or of dissolved titanium was speculated. The quantity in the tissue was below the 30 parts per million detection limit of the imaging technique.^50^ In another study following administration of agglomerates of 7 nm and of 10–25 nm amorphous SiO_2_ NPs to rats in their feed for 28 or 84 days, absorption through the GI tract to peripheral tissues was less than 0.25%, and may have occurred from the absorption of either intact particles and/or of silicic acid released from the particle surface.^51^, ^52^ The percent absorption decreased as dose increased, likely due to gelation of the silica particles into agglomerates in the lumen at the higher doses.

The mechanisms suggested by the TiO_2_ and SiO_2_ data include a combination of transcellular transport of intact particles via phagocytosis through M‐cells of Peyer's patches, transcellular and paracellular transport of disintegrated molecules released from the particle surface, paracellular transport of the intact particles between damaged epithelial cells, and persorption of intact particles through dead epithelial cells.^50^, ^53^, ^54^

As research progresses, data on the full range of novel chemical entities that may be proposed for use in food will emerge.

## GASTROINTESTINAL LUMINAL FACTORS THAT INFLUENCE THE SIZE AND SURFACE PROPERTIES OF NMs

The absorption of an administered NM through mucus and epithelial cells is a function of its size, aggregation, shape, surface properties, and surface corona. All of those physicochemical properties can be impacted and modified by interaction with factors encountered in the various environments of the luminal milieu during transit through the compartments of the mammalian GI tract (Figure 1). Hydrophobic (lipid soluble) particles are generally considered to be more readily absorbed than hydrophilic (water soluble) particles.^55^ However, there are exceptions. A hydrophilic polyethylene glycol coating has been shown to enhance stability and bioavailability *in vivo* during digestion.^56^ Some carbohydrate‐binding plant lectin proteins such as wheat germ agglutinin have affinity for receptors on intestinal enterocytes, facilitating transport across the cellular barrier when incorporated in solid lipid NPs.^57^ Particle surface charge properties can also affect adhesion with the negatively charged mucus layer and thus exposure to intestinal cells.^28^ While mucoadhesion may trap a NP and limit its positioning for epithelial absorption, it can increase residence time and protect from digestion.^58^ Along with the effects of inherent surface properties, particle size is a key factor in determining mucus transit and cellular uptake.^12^, ^59^ Smaller NPs can more easily diffuse through the mucus network pores and gain access to cells. Permanent degradation, dissolution and/or aggregation during digestion can however eliminate any such nanosize specific outcomes. Various parameters in the different compartments of the GI tract can play a role in influencing digestion and absorption.

We reviewed the modifying role of the major physicochemical factors in saliva, gastric fluid, and intestinal fluids: physical forces, osmotic concentration, pH, digestive enzymes, other food and endogenous biochemicals, and commensal microbes. In conjunction, *in vitro* and *in silico* data were assessed for evidence that these factors can have effects on the integrity, aggregation, and surface properties of metal, mineral, carbohydrate, nucleic acid, protein, and lipid primary nanostructures, with a focus on specific examples of TiO_2_, SiO_2_, and cellulose NMs.

### Physical Forces from Chewing and from Peristalsis during Transit

Mastication forces in the buccal cavity break down solid food matrices, resulting in a polydisperse fragment size distribution.^60^ Tongue forces mix the food with salivary fluid secreted from the parotid and salivary glands. Fragments with an average diameter smaller than approximately 2 mm can then be swallowed and pass down the esophagus to the stomach.^61^ Peristaltic waves resulting from contraction of muscles in the GI tract wall propel this digesta forward. In the stomach peristaltic forces are generally in the range of 5–20 mmHg, and occasional wave contractions can be as strong as 150 mmHg.^62^ These forces break down the digesta to at least the 1 mm diameter before passage from the stomach to the small intestine.^60^ The transit time of chyme is on the order of hours in the stomach and up to several days in the intestines.^63^, ^64^ Engineered NMs released from food matrices during this transit may be impacted by the physical forces they encounter.

To date, direct published studies were not identified on the modification of NP primary particle size distribution and aggregation from physical forces in the range of those encountered during chewing and intestinal peristalsis. However, an example of indirect *in vitro* fluid incubation data suggests that peristalsis‐level mixing forces can mildly modify the degradation and aggregation of cellulose NPs in a colloidal suspension.^65^, ^66^ There is a data gap on the effect of stronger forces equivalent to chewing on these and other categories of food‐relevant NPs.

### Osmotic Concentration and pH

Fluid volume and solute concentration are highly variable in the GI tract compartments and fluctuate in response to ingested food.^67^, ^68^ High solute ionic strength can modify the dissolution and precipitation behavior of NMs via the salting out effect. Differently charged ions can also modify particle surface charge and zeta (ζ) potential and, therefore magnitude of electrostatic repulsion. Conversely ions can act as sandwich‐filling elements, allowing equally charged species to interact. Together these modifications of surface properties determine the growth and size of NM agglomerates.^4^

pH‐related environments along the GI tract change from the oral cavity to the rectum and from the lumen through the mucus layer. Saliva has a neutral pH of approximately 7.^69^ Although the pH in the stomach can be as low as 1, NPs in food are likely to be buffered by the food matrix, and are thus likely to be exposed to pH values in the range of 2–6 when consumed in a meal.^70^ The pH gradient of the mucus varies from as low as 1 in the lumen of the stomach to nearly neutral at the epithelial surface.^71^, ^72^ In the small and large intestine lumen the pH ranges from 5 to 8.^73^ Therefore food related NMs encounter a fluctuating pH spectrum while traveling down the GI tract.

With regard to ionic strength and pH, direct *in vitro* fluid and *in silico* computational evidence reflective of conditions encountered in the mammalian GI tract has demonstrated that these parameters have major impacts on the surface charge and aggregation of the reviewed NPs. For example in a fluid suspension, increasing the sodium chloride concentration suppressed surface electrostatic repulsion of TiO_2_ NPs, allowing van der Waals interactions to manifest, and the rate of aggregation to increase.^74^ Likewise divalent cations in water adsorbed to the surface TiO_2_ NPs, neutralizing their negative charge and inducing aggregation.^75^, ^76^ Similarly the theoretical inter‐NP hydrogen bonding potential bridging the surface of colloidal amorphous SiO_2_ NPs in an aqueous solution was calculated to be modified by the concentration of calcium and chloride ions.^77^ Nanocellulose fibrils also aggregated as ionic salt concentration increased, and they aggregated as pH decreased, as a result of reduced surface charge in both cases.^78^, ^79^ Increasing the pH modified the surface charge of TiO_2_ NPs, and therefore the rate of aggregation, the size of the aggregates and the rate of sedimentation.^76^, ^80^, ^81^, ^82^ The pH conditions are also particularly important for solid metalloid‐based NMs such as clay, as acid can partially or completely solubilize the particles and in turn the minerals can act as buffers, influencing local pH values.^83^

*In vivo* the various GI parameters are dynamic, and thus can be simulated in static or dynamic *in vitro* GI models to determine how NPs will change during transit through the various compartments. For example, in a simulated salivary fluid SiO_2_ NPs administered in water and food matrices were present as single particles and as small aggregates.^84^ In a gastric digestion solution containing the mammalian digestive enzyme pepsin at pH 2, TiO_2_ had a positive surface charge whereas SiO_2_ was neutral and agglomerated to significantly larger clusters.^84^, ^85^ Following subsequent simulated intestinal digestion in a solution containing of a mixture of digestive enzymes at pH 7 followed by a solution containing bile salts at pH 7, both materials were negatively charged and the SiO_2_ deagglomerated back to individual particles in the nano size range. The aggregation in the stomach fluid was attributed to the low pH and high ionic strength, but not to the activity of the enzymes. Clearly osmotic concentration and pH have a major impact on the surface and size of NPs.

### Digestive Enzymes

The effects of endogenous enzymes such as buccal amylase, gastric pepsin, and intestinal pancreatic lipase and nucleases can have an influence on the integrity of some categories of ingested particles. For example, ‘soft lipid’ or ‘solid non‐lipid non‐metal’ biochemical macromolecule‐based NMs may be susceptible to digestion. Mammalian enzymes can also denude surface‐adsorbed biochemicals from stable particles, but re‐adsorption of novel entities will occur.^4^

Inorganic compounds are generally not the substrate of mammalian enzymes. Therefore the digestive impact on that category of particles is generally not studied as an independent variable.^84^, ^85^ However NPs composed of starch, lipids, protein, or nucleic acids can be influenced by digestive activity.^86^, ^87^

### Endogenous Biochemicals and Food Matrix Biochemicals

Particles in the GI milieu are exposed to a range of endogenous and ingested biochemicals and surfactants that can reversibly adsorb to the particle surface, forming a corona that changes the size and surface properties.^88^ For example when emulsion particles consisting of soy oil coated by β‐lactoglobulin protein in water were exposed to artificial saliva, free mucin proteins in the mixture bridged the particles and caused them to flocculate out of suspension.^89^ Adding plant pectin protein to the coating of these emulsion particles increased their electrostatic and steric repulsive forces and stabilized the emulsion.

Bile secreted into the small intestine contains bile salts and phospholipids.^90^ Bile salts are among the most surface active components in the intestine, and affect colloidal behavior. They facilitate the solubilization of free fatty acids, released from triglycerides during digestion, into submicron sized mixed micelles prior to absorption, and can similarly solubilize NMs.^25^ For example bile salt adsorption to 500 nm emulsion droplets that were composed of bovine milk protein, caseinate, and medium‐chain triglyceride oil has been shown to significantly increase diffusion through purified intestinal mucus.^24^, ^91^ The negative charge imparted by the bile salt significantly reduced the adhesive electrostatic interactions with the negatively charged mucus network. Thus, the integrity and uptake of ‘soft lipid‐based’ NMs can particularly be modified. These surfactants may similarly bind to non‐lipid NPs, modifying their size, agglomeration, and properties.

Phospholipids are not only endogenously present in gastric secretions and in intestinal bile, but also enter the GI tract as components of food, which results in a variable concentration throughout the canal.^92^ These zwitterionic surfactants contribute to the solubilization of especially lipophilic compounds by decreasing the surface tension. Similarly other endogenous and dietary organic molecules including lipids, proteins, nucleic acids, carbohydrates, and dietary fibre can adsorb to the particle surface with binding strength dependent on pK and stoichiometry. There are various examples.

In aqueous fluids, exogenous surfactants and organic matter controlled size of TiO_2_ NP and carboxymethylcellulose NP aggregates.^66^, ^74^, ^80^ A corona covering composed of bile salts and/or proteins developed on the surface of TiO_2_ and SiO_2_ aggregates during incubation in simulated intestinal juices.^85^ In another study SiO_2_ NPs formed large agglomerates in intestinal fed matrix conditions, but not in intestinal fasted, gastric fed, or gastric fasted simulations.^93^ Combining SiO_2_ NPs with a low fat coffee creamer food matrix prevented their aggregation compared with incubation in water alone.^94^ Clearly the binding of biochemicals found in the GI lumen can greatly modify the particle surface and size.

### Commensal Microbes

The GI tract harbors an extremely complex microbiota that participates in digestive function and is important in homeostasis and gut‐associated immune function.^95^, ^96^, ^97^ Next‐generation sequencing techniques with samples from the human gut have identified approximately 1000 different bacterial species.^98^ Bacteria do not have active endocytic mechanisms to take up larger particles.^99^ However, adherence of particles to persistent microbial biofilms in the oral and large intestine environments can occur, and microbial secretions can interact with NPs.^100^, ^101^

Relatively few studies have investigated the influence of cultured microbes and their secreted fermentation enzymes on NP integrity. Cellulase enzyme purified from anaerobic bacteria and from fungi was shown to partially digest the nanoscale architecture of cellulose microfibers.^102^, ^103^ However, this effect is specific to particles composed of that biochemical substrate. The addition of SiO_2_ content within carboxymethyl cellulose particles significantly reduced their hydrolysis by cellulase.^104^ The impact of microbes on other categories of NPs, and any reciprocal effects on the microbes, are key areas for further research.

The complexity of GI factors is increased further when we consider the natural differences that exist between individuals.

### Physiological Variability and Diseases

Differences in normal physiology as well as specific diseases present altered GI environments and affect epithelial permeability. Therefore it is important to consider how such inter‐individual differences may affect the stability and movement of orally administered substances.^53^

The age of an individual can affect a variety of factors such as the pH at each point of the GI tract, the transit time of fluids, and the barrier function of the lining. In infants, permeability is significantly higher than in the average adult. Old age can also affect GI functions, resulting in decreases in acid and enzyme secretion, digestion, motility, and nutrient absorption.^105^

Gender‐specific differences can exist. In male rats for example, spherical 26 nm TiO_2_ NPs orally administered by gastric gavage for 90 days significantly increased the level of titanium in blood to approximately 1.25‐fold of the background levels in non‐exposed rats.^106^ There was no significant increase in females.

Pregnancy causes physiological changes in the digestive system.^107^ In addition, pregnancy can have common complications such as gastroesophageal reflux disease, peptic ulcer disease, inflammatory bowel disease, and irritable bowel syndrome. These conditions may affect the behavior and uptake of NMs, e.g., those developed for uses in food supplements to increase nutrient uptake, ease of digestion, or bioavailability.

Malnutrition greatly impacts GI physiology and structural aspects.^108^ The sleep cycle also affects digestive function, and stress at high levels can increase inflammatory responses and thus contribute to increased GI lining permeability.^109^

Inflammatory Bowel Disease is a common GI condition, which includes Crohn's disease and ulcerative colitis. The abnormal mucus layer and inflamed tissue associated with these conditions has been shown to result in increased susceptibility to the absorption of some types of NMs. For example in a mouse model of ulcerative colitis SiO_2_ NPs adhered to ulcerated regions of the inflamed tissue at a sixfold higher percentage than to non‐inflamed healthy tissue.^110^ A retrospective microscopy study with lymphoid aggregate and Peyer's patch tissues surgically resected from normal areas of the small and large intestine of human volunteers with Crohn's disease, ulcerative colitis and colonic carcinoma observed titanium oxide particles of 100–200 nm in diameter, as well as 100–700 nm particles consisting of silicon, magnesium, potassium, sodium, and iron, in phagolysosomes in macrophage cells underlying the epithelium.^111^ The authors postulated the source to be food additives and environmental exposures.

There can also be abnormal GI barrier function in Celiac disease and other autoimmune diseases.^39^, ^112^ Likewise gastroenteritis can increase gut permeability, for example following infection with *Campylobacter jejuni*, enteropathogenic *Escherischa coli,* or *Clostridium difficilie*.^109^, ^113^ The infection of human intestinal biopsies with *Yersinia pseudotuberculosis* significantly increased the transcellular macropinocytosis of fluorescent 200 nm and 500 nm polystyrene particles.^114^ GI pre‐cancerous lesions, tumors, and cancer treatments can also reduce gut barrier function.^115^

Clearly certain human subpopulations have differences in GI physiology that should be accounted for in the assessment of the trafficking of NMs.

## CONCLUSIONS

The mammalian GI tract efficiently processes the chemical constituents and structures of food ranging from bulk materials down to atoms during transit through the buccal cavity, stomach, small intestine, and large intestine. Permeation of particulate matter through the buccal cavity epithelium is understudied, likely because the small intestine has the highest surface area and specialization for nutrient uptake. Intestinal enterocytes are the most abundant cell type in the intestinal epithelium and thus the most important barrier to absorption. M‐cells over GALTs make up less than 1% of the intestinal surface area, but are the cell type most specialized for the uptake of particulate matter. Mucus coating the epithelium is also an important barrier to absorption.

Novel engineered NMs have many promising beneficial applications in food and food contact materials. In some jurisdictional legislations those applications may require a safety assessment, particularly if the bioavailability has changed compared with a conventional bulk form. Size is a key characteristic in the bioavailability of NMs. The physicochemical properties of the NM surface also affect stability, agglomeration, interactions with mucus, interactions with the apical cell membrane, absorption, and excretion. If *in vitro* cellular, *in vitro* fluid or *in silico* computational models are to be adapted to accurately predict human GI digestion and absorption of novel food‐relevant engineered NMs, it is important to know which parameters are essential to incorporate in these models. Direct and indirect evidence confirms that salt concentration, pH, and biochemicals in the luminal fluid matrix are key in determining the integrity, aggregation, and surface properties of food‐relevant NPs, and therefore important in determining their absorption into systemic circulation. Physical forces, digestive enzymes, and microbes may also have impacts.

Further research is required to fill data gaps on the kinetics and absorption of the full spectrum of potentially food‐relevant NMs (Table 1). Improving our understanding of the relationship of the physicochemical aspects of NMs with the GI ecosystem will help provide data useful for risk assessment. Interlaboratory validation studies can lend strength to methods development and findings. In particular the models of the GI tract outlined as applicable by the NanoRelease Food Additive project will be useful for this purpose.^13^ Filling methodological gaps will allow comparison of NMs with the ionic and bulk conventional forms of chemical additives in food. Subsequently, data on the distribution of the NMs in the body along with their metabolism and excretion will complete the toxicokinetic analysis. International risk assessment of novel engineered NMs is underway as exposure, toxicokinetic and toxicology data on other key endpoints becomes available. The development of methodologies to facilitate the detection of the full range of food‐relevant NMs will facilitate risk management and public policy.

**Table 1:** Knowledge Gaps on Mammalian Gastrointestinal Tract Digestive Parameters Modulating the Integrity, Surface Properties, and Absorption of Food‐Relevant Nanomaterials

Knowledge Gap Questions
Among physical forces, osmotic concentration, pH, digestive enzymes, other biochemicals, and commensal microbes, which GI luminal parameters are the strongest inducers of any changes observed in the size, shape, surface properties, and surface corona of NMs?
Is the size, shape, and surface properties of the full range of potentially food‐relevant NMs modified in the GI luminal milieu, or only certain categories of NMs?
What is the difference in percent absorption through the GI tract epithelium of NMs with different physicochemical properties?
Through which GI tract organs and epithelial cell subsets does the absorption of NMs of different chemical makeup occur?
What inherent properties of NMs of different chemical makeup determine their percent absorption through mucus and epithelial cells?
Does the percent absorption of a given NM differ from the mass‐balanced percent absorption of the bulk or ionic form of the same chemical?
